# Artificial Intelligence's Potential in Zoo Animal Welfare

**DOI:** 10.1002/zoo.70008

**Published:** 2025-07-14

**Authors:** Matyas Liptovszky, Emily Polla

**Affiliations:** ^1^ Life Sciences Directorate, Perth Zoo South Perth Western Australia Australia; ^2^ The Animal Welfare Science Centre, Faculty of Science The University of Melbourne Parkville Victoria Australia

**Keywords:** AI, animal wellbeing, behavior, machine learning, monitoring

## Abstract

The thorough, objective, and regular assessment of animal welfare in zoos and aquariums is rapidly becoming an essential task for these institutions. Traditional welfare assessment methods are, however, difficult to scale to the number of species and individuals housed in zoos and aquariums. Automation, using artificial intelligence (AI) can provide solutions to these challenges. This literature review provides an overview of recent advances in this field, with a focus on studies relevant to zoo and aquarium animal welfare. AI in animal behavior and welfare monitoring, particularly in farm animals, has become increasingly commonplace in recent years. Recent studies have investigated AI's capability to identify and assess animal behavior in poultry, pigs, sheep, and cattle, including estrus prediction in cows; classification of animal vocalizations; and detection of potential welfare concerns, including early signs of lameness in cattle and sheep. In companion animals, AI has been used for facial recognition, vocalization‐based emotion recognition, and behavioral monitoring. Laboratory animal behavior monitoring through AI tools has also rapidly increased since 2000. AI is increasingly used in zoos, including the identification of individual animals; monitoring of their movement within their enclosure; and quantifying behavior, including time spent using enrichment. The rapid increase in AI use in animal welfare shows promise in improving animal management and welfare in zoos and aquariums, through improved and more efficient monitoring and prediction.

## Summary

- Artificial intelligence (AI) describes a computer system that can adapt to changing environments and feedback loops.
- The use of AI in animal welfare has increased in recent years, including in zoos and aquariums.
- AI is rapidly becoming accessible and affordable for various use cases, including for animal welfare.

## Graphical abstract

Heat map visualization of enclosure space use by meerkats, generated by the artificial intelligence model described in the case study.

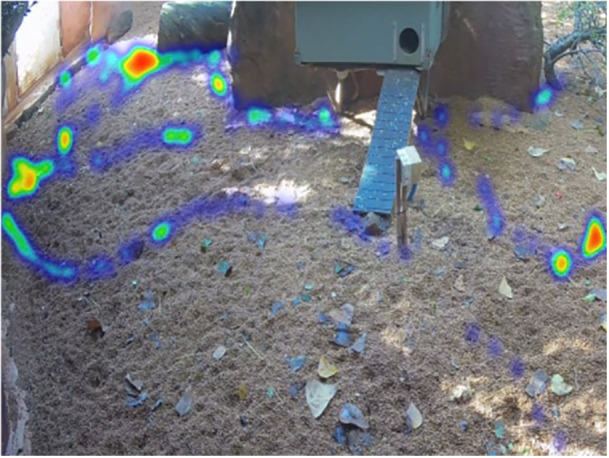

## 1 Introduction

Animal welfare underlines everything modern zoos and aquariums do, as their social license to operate relies on ensuring positive welfare outcomes for their animals. This is reflected in the exponential growth of zoo animal welfare research in recent years (Walker et al. 2014; Binding et al. 2020). However, increasing demands for animal welfare monitoring, including assessments of physiological and behavioral signs of positive and negative affective states, have created resource challenges for these organizations. Traditionally, animal welfare assessments in zoos focused on input or resource assessments, as these were more practical to achieve, though often provide an incomplete picture. Assessing output or animal measures using quantitative data offers better insights, but can be time‐ and labor‐intensive (Manteca et al. 2016; Jones et al. 2022). This reliance on manual data collection and analysis potentially limits the scope and depth of welfare assessments.

Big data approaches offer a more holistic perspective on animal welfare by integrating multiple metrics for comprehensive analysis. Zoological institutions have been collecting vast amounts of rich data on the animals in their care for decades, including animal husbandry, nutrition, and veterinary records. For example, the Species360 Zoological Information Management System (ZIMS) contains vast datasets on about 10 million animals of 22,000 species kept in over 1300 zoos and aquariums (Species360 Zoological Information Management System ZIMS 2024). However, the sheer size and complexity of such data (also called “big data” [Favaretto et al. 2020]) make it difficult to analyze it effectively using traditional methods.

Artificial intelligence (AI) offers a promising solution to these challenges. Since the invention of the term in 1956 (Kaul et al. 2020), AI grew from a vague theoretical concept to technology commonly utilized in our everyday lives. It is now present in most smartphones, smart speakers, televisions, and many other common electronic devices, but it is also utilized in manufacturing, healthcare, commerce, education, and agriculture (Bini 2018). It has inherent benefits in analyzing large and complex datasets fast, freeing up human resources. In addition to more traditional computer data sources, AI is now used to analyze still and motion pictures; sound, including human speech; and text. These open opportunities for endless different applications, from computer vision, speech to text, translation, or transcription services to more bespoke expert systems to provide medical diagnoses, financial, or weather forecasting.

It could also support a more holistic approach to animal welfare assessment, providing an overall analysis of many different measurements, taking a “big data” approach (Liptovszky 2024). AI assisted data mining (Lakshmi and Raghunandhan 2011) of large datasets could create new knowledge of zoo animal welfare.

This paper reviews the currently available literature about AI in animal welfare, with a focus on zoo and aquarium animal welfare. Research published on AI use on animal welfare in zoological institutions is growing, though currently tracks behind farm, companion, and laboratory animal studies. It is, therefore, important that the zoo and aquarium community remains up to date with the development of this rapidly evolving field, to utilize the potential benefits of it in the future.

## 2 AI and Related Fields

Despite the term being used for more than six decades, AI remains challenging to define. There are several different attempts to pinpoint its characteristics, and it is both described as nonliving agents that act intelligently or rationally (in the mathematical sense of the word) or nonliving systems which are comparable to humans in intelligence. They can also be considered as computer systems, which can not only follow simple, predefined rules, but also capable of adapting their operations to changing environments and feedback loops and maximizing their chances to achieve a task (Russell et al. 2010).

Machine learning (ML) is a subset of AI, in which a computer software (or code) is developed in a way which enables the software to “learn,” and to adapt to achieve increasingly better outcomes (Appleby and Basran 2022). Some ML algorithms (also called supervised learning) require “training” data paired with corresponding labels (i.e., correct identification of the data), enabling the trained model to extrapolate classification or predictions to large datasets. In other cases (called non‐supervised learning), the training data set is not labeled, and the ML model is designed to extract features from the data. This can be used to classify or group similar elements of the data (Jiang et al. 2017).

A further subset of ML, artificial neural networks (ANNs) seemingly mimic the function of the neurons within a biological brain. Artificial neurons, like synapses in the nervous system, connect to each other and transmit information through several connections (Appleby and Basran 2022; Montesinos López et al. 2022). In these systems, information travels from the input layer towards the output layer, passing through at least one hidden layer. The power of these models lies in the large number of simple processing units, which can operate parallel to each other. Deep learning is the name of an ANN with multiple hidden layers, in which different layers might perform different transformations of the data. The term “deep” in this meaning refers to the depth crated by multiple layers, rather than some form of deep knowledge (Montesinos López et al. 2022).

Different AI algorithms are frequently used in applications like machine vision, speech recognition, or working on medical diagnoses. Examples of practical applications of these methods are Google's Translate natural language processing (NLP) and translation, Apple's Siri or Amazon's Alexa human voice recognition, or IBM's Watson Health medical AI solutions (Bini 2018).

## 3 Current Use Cases of AI in Animal Welfare

### 3.1 Farm Animals

There is strong interest in AI in farm management from an economic perspective, with increasing efficiency and decreasing costs (including personnel costs) being key drivers. Precision Livestock Farming (PLF, for a recent overview, see Kleen and Guatteo [2023]) aims to utilize connected sensors and controls (also called Internet of Things), big data, and ML algorithms to automate animal monitoring and management processes. Sensors and data from these can include temperature, humidity, air quality, and other environmental parameters, still or motion picture cameras, individual identification of animals (e.g., through radio‐frequency identification), accelerometers and location sensors measuring and controlling animal location, movement, and space use, as well as other sensors, including more involved physiological monitoring or output parameters, like milk production (Yaseer and Chen 2021). Data generated from many sensors can be analyzed and used for various purposes, including disease monitoring and preventative health, animal behavior monitoring and linked predictions, or monitoring and predicting animal production. Visual monitoring can support individual animal identification through facial or other pattern recognition, or more specific analysis, including body condition scoring or gait analysis and lameness detection (Yaseer and Chen 2021). In broiler chickens, complex tasks, like predicting growth and body weight, presence or absence of ascites, or separating healthy animals from those showing clinical signs of avian influenza by AI are already achieved at exceedingly high (98%–100%) accuracy (meaning the ratio of all correct (positive and negative) predictions from the total number of cases examined; further see Hicks et al. (2022) and Milosevic et al. (2019).

A systematic literature review of AI in animal farming has been completed recently, identifying significant increase in publications in this area between 2016 and 2021, compared to the previous period (Bao and Xie 2022). Animal behavior analysis and recognition was the most frequent topic of studies, while pig (38%), cattle (37.5%), and poultry (17%) were most represented species in the reviewed 131 research papers. Another study (Benos et al. 2021) reviewing 338 papers between 2018 and 2020 on all agricultural applications found that cattle, sheep, and goat are currently the most studied animals, followed by pig and poultry. In terms of technology, motion capture sensors were the most studied, followed by visual and audio sensors, physical and growth characteristics, and weather data. While there was rapid increase found in the number of publications over a 3‐year period, livestock management studies grew slower than studies focusing on crop management.

Many recent studies investigated AI's capability to assess animal behavior. A key underlying concept to detect and measure animal behavior from motion pictures is pose detection (for an overview, see Mathis et al. [2020]). Pose detection refers to the process of identifying the position of different body parts (i.e., head, legs) of an individual animal. Deep learning methods are well‐suited for this task both for single‐ and multi‐individual situations (Nath et al. 2019; Fang et al. 2021), therefore, it's not surprising that pose detection and based on that behavior detection and measurement received strong attention in recent years. Recent studies include body, head, and tail posture in pigs (D'Eath et al. 2018; Ocepek et al. 2022), detection and classification of different poses and behaviors in sheep, cattle, and chicken (Rahman et al. 2018; Walton et al. 2018; Fang et al. 2021), and prediction of estrus and calving day in cattle based on behavior (Keceli et al. 2020; Wang et al. 2020).

Detection of early lameness in cattle and sheep (Kaler et al. 2020; Taneja et al. 2020; Warner et al. 2020), or mastitis in cattle (Hyde et al. 2020), and early prediction of Coccidiosis in poultry (Borgonovo et al. 2020) are current examples of health monitoring applications in farm animals.

### 3.2 Companion Animals

While companion animal applications of AI are clearly behind those of production animals, recent developments demonstrated its use, primarily in dogs. Domestic dog face recognition is already possible with good reliability. In one study, the system was able to identify individual animals from 48 different dogs with 88% accuracy, based on a single image. The model was also able to cluster pictures of unknown dogs (Mougeot et al. 2019). The ability to reliably identify individuals is important in zoo animal welfare, where we typically work with a smaller number of individuals per species, compared to free‐ranging wildlife or farm animals. Being able to identify individual differences in behavior, diet consumption, health, or environment use could enhance our understanding of the welfare of such individuals and ensure appropriate measures are taken to maximize that.

Speech‐based emotion recognition in humans is well developed, and some of the underlying principles seem to be transferable to other mammalian species. Built on this, classification of dog barks is another area under development currently. Barks have context and individual specific features, and an AI model was able to categorize 6000 barks recorded in six different behavioral situations with 43% and 52% accuracy, respectively (Molnár et al. 2008). Another study (Hantke et al. 2018) in dogs focused on context and perceived emotional classification, as well as perceived emotional intensity using different AI models. Emotions investigated were aggression, despair, fear, fun, and happiness. The authors concluded that due to physiological and psychological similarities, different emotions create similar acoustic characteristics across different mammal species, therefore, human speech‐based emotion recognition is a viable pathway for other mammal species as well. ML models have also been utilized, considering physical and physiological metrics to determine feed estimation to help prevent companion animal obesity (Ravi and Choi 2022).

Camera and sensor‐based behavioral monitoring systems are another field where AI is becoming more established in companion animals, achieving high accuracy levels for both walking (99.5%) and resting pattern recognition (97%) (Boteju et al. 2020). Breed recognition in the same study, however, achieved lower accuracy levels (89% in a two‐way choice). Facial expression recognition has also been utilized in horses and cats to assess pain (Lencioni et al. 2021; Feighelstein et al. 2023), a method which is well‐described for pain scoring using grimace scales (Dalla Costa et al. 2014; Evangelista et al. 2019).

### 3.3 Laboratory Animals

AI applications for the analysis and measurement of laboratory animal behavior have been used as early as 2000, when automated posture classification was proposed using neural networks (Heeren and Cools 2000). Since then, automated tracking and behavioral analysis of single and multiple rodents have been resolved, including measurement of social behavior (Giancardo et al. 2013; Hong et al. 2015; Bohnslav et al. 2021; Isik and Unal 2023). These models, including some open‐source (freely available) software, can have direct relevance to zoo and aquarium settings as well. Multi‐individual behavioral tracking might be especially important in aquariums, where large number of individuals can pose logistical challenges for human observers.

Further to the monitoring of normal behavior, Weber et al. (2022) provide an example of monitoring the recovery of rodents following neurological disturbance (stroke, in this instance). The authors suggest their method might also be useful to monitor other disease processes which impact locomotion.

While automated analysis of video sequences is now relatively common, the real time monitoring of animals, and their behavior is of great interest for animal welfare purposes. This is a challenging task, given the computational resources required by most machine vision systems. Cocoma‐Ortega et al. (2022) described a system run on inexpensive hardware, capable of real‐time detection of a rat in an open maze, as well as monitoring of the rat's behavior. In their two‐step process, the system first identifies the rat in the image with a precise location, then a downscaled version of the image is passed on to a behavior analysis module, therefore decreasing compute requirements. Ambiguous behaviors were recognized with an average 60% precision (meaning the ratio of correctly classified predictions and the total number of cases classified in that group; further see Hicks et al. [2022]). Multi‐animal pose tracking has also been proposed, and tested in flies, bees, mice, and gerbils, achieving real‐time speeds (Pereira et al. 2022). Another open‐source model tested multi‐animal pose estimation and tracking on the fish species inland silverside (*Menidia beryllina*), mice, and common marmosets (Lauer et al. 2022).

Kahnau et al. (2023) provide a review of home cage monitoring in laboratory rats and mice, which might be of interest to zoo and aquarium professionals. They found that manual monitoring has been gradually replaced by automated methods since the 2000s, and currently these automated systems are mostly used to monitor locomotor activity, feeding, and social behaviors.

The main limitation of these examples is that laboratory animal environments, especially under experimental conditions, are much less complex than typical zoo and aquarium environments. Therefore, we must be cautious about expectations of the applicability of these models directly to other species in a different environment. However, these examples demonstrate current AI capabilities, as well as provide starting points, including open‐source software in many instances, for developing zoo and aquarium specific models.

### 3.4 Zoo, Aquarium, and Wild Animals

The use of AI in zoo and wild animals is growing, but primarily driven by free ranging wild animal applications at this stage (for recent reviews see Lamba et al. [2019]; Isabelle and Westerlund [2022]; Tuia et al. [2022]). Processing large volumes of camera trap data from field surveys pose a significant challenge to scale up these projects; therefore, it is understandable that these applications have been prioritized. As outlined above, AI is well suited for these tasks, including identification of species, individuals, body positions, and behaviors (Deb et al. 2018; Schofield et al. 2019; Schütz et al. 2022; Tuia et al. 2022; Bendel and Yürekkirmaz 2023; Gerdan Koc et al. 2024; Paulet et al. 2024). Machine vision has also been used to assess population health of whales through body length and condition measurements (Bierlich et al. 2024).

A systematic review (Diana et al. 2021) examined the use of technology to support animal welfare assessment in zoo animals, revealing significant gaps compared to similar research in livestock. Though this was not specific to AI, the reviewed papers are relevant for our topic. Of the 19 studies identified, most focused on individual monitoring using cameras (52.6%) and wearable sensors (31.6%), primarily measuring behavior (63.1%) or physical/physiological parameters (31.6%), with limited attention to environmental factors. By contrast, livestock studies (e.g., pigs) often utilized detached sensors for group‐level monitoring, indicating a technological disparity. Mammals were disproportionately represented (89.5%), especially elephants and primates, while reptiles, lower vertebrates, and invertebrates were largely neglected. Most zoo research used technology for enrichment rather than continuous, automated monitoring, the latter of which is common in PLF. The authors emphasized the need for integrated approaches combining multiple parameters to improve welfare assessment and highlighted significant potential for advancing automated welfare monitoring in zoos.

Individual identification is a focus area of current research on AI and technology applications in zoos, as typically, automated tools need to differentiate between individual animals to enable meaningful data analysis. Facial recognition software, for instance, has demonstrated 97% accuracy in identifying individual gorillas in zoo environments. Although this approach requires significant interdisciplinary collaboration and investment (Brookes et al. 2022), it enables automated tracking of enrichment device usage. The data reveal individual engagement levels and duration, insights previously obtainable only through labor‐intensive observations or video analysis. Individual identification has also been successfully achieved in all eight bear species in captivity (Chen et al. 2020; Clapham et al. 2022).

Zuerl et al. (2022) developed an automated, video‐based framework for behavioral monitoring in zoos, which surpasses traditional observation methods. Their system identifies individual polar bears with 86.4% reliability and achieves a localization accuracy of about 20 cm. It analyzes spatio‐temporal enclosure use, individual activity patterns, social proximities, and area preferences. The framework is designed to handle challenges typical in zoo environments, such as large enclosures, variable camera angles, low resolutions, and changing lighting conditions, making it adaptable across species using a generic approach. The authors highlighted that most existing methods fail to fully automate manual observations, a gap their framework addresses.

Congdon et al. (2022) presented an AI model originally designed for humans, adapted for Sumatran orangutans. Using five 12‐megapixel cameras installed in the orangutan habitat, the system provided comprehensive coverage and minimal blind spots. The collected images were used to train a deep neural network for orangutan identification and behavioral analysis. The model achieved 95% accuracy in species detection and 80% in individual recognition from a single image, rising to 92% with 50 images. This technology can support early detection of health or welfare changes and inform husbandry improvements. Gammelgård et al. (2024) describe a similar, albeit less capable model in Bornean orangutans, which performed well in individual identification, but was less capable of distinguishing between behavioral categories. A proof‐of‐concept study also demonstrated the ability of ML to analyze thermoregulatory behavior on captive Rough‐tail rock agama (*Laudakia vulgaris*). While originally this study was developed to demonstrate AI's potential to streamline ecological monitoring, insights into species' thermal preferences would be highly relevant in zoo environments as well.

Understanding the welfare impact of zoo activities is central to improving zoo and aquarium animal welfare. Pertoldi et al. (2024) compared ML and traditional methods to evaluate visitor effects on black lemurs (*Eulemur macaco*) and ring‐tailed lemurs (*Lemur catta*). They observed minor differences between the methods, concluding that ML and traditional observations can be complementary.

Information on animal behavior, however, can also be acquired without video monitoring. Jeantet et al. (2018) tested the effectiveness of accelerometers in identifying sea turtle behaviors by attaching sensors to a loggerhead (*Caretta caretta*), a hawksbill (*Eretmochelys imbricata*), and a green turtle (*Chelonia mydas*) in a public aquarium setting. Data from the sensors were matched with video recordings, and two ML methods were used to classify behaviors. Accuracy reached up to 87% depending on the method and species, demonstrating the viability of this technology, when other monitoring methods might be challenging.

## 4 Future Directions

Through analyzing the currently existing literature on AI in animal welfare, it is evident that in the future, applications will become more complex, being able to analyze a multitude of data sources to provide a better assessment of welfare, compared to the current focus on more specific, but narrowly focused technologies, like individual animal recognition based on images. Big data, primarily based on automatic data collection from a range of sensors, can provide a huge step forward in the assessment of animal welfare, compared to the current methodologies (Liptovszky 2024). Real‐time or near real‐time assessment of the animals' behavior and response to stimuli, and automated affective state recognition might all become reality, providing insight animal welfare scientists have not had before (Neethirajan 2022). Early detection of distress, fear, and other negative experiences can lead to early interventions from animal care staff, preventing further negative welfare states, reduced health, or other impacts requiring veterinary interventions (Neethirajan 2021). Using AI to measure body length and condition, where possible for specific species as demonstrated in a wildlife study, may benefit zoo animal health monitoring, and inform other welfare‐related factors, such as the impact of diet on animal growth rates (Bierlich et al. 2024). Monitoring of animal gait and mobility through AI could benefit animal welfare decisions surrounding quality of life, particularly in geriatric animals with conditions such as arthritis.

There is great scope in integrating animal behavioral and physiological data with environmental data. Current applications commonly focus on a single approach, primarily on the former, missing the opportunity to create a deeper understanding of the interaction between environmental parameters and animal behavior and welfare (Diana et al. 2021). Precedents for this approach can be found in farm animals, where a combination of environmental sensors and automated environmental controls are commonly used.

Developing and testing technologies for nonmammalian species also appear to be a priority for the future, given the current narrow focus on mammals, and even within that, on a few high‐profile taxa, like elephants and primates (Diana et al. 2021). Zoo animal welfare research shows a persistent and significant bias towards mammals, as highlighted by multiple studies. A review of literature from 2008 to 2017 found that mammals accounted for 75% of studies, with vertebrates comprising 82%, and great apes being particularly prominent (Binding et al. 2020). Long‐term trends are consistent with this, with a bibliometric analysis (1966–2007) showing 75.92% of experimental studies centered on mammals (Goulart et al. 2009).

While the current scientific literature is primarily focused on new ways of data collection, management, and analysis, examples from other fields, including the business world, are suggestive, that equally significant changes can be expected in how data are interpreted, visualized, and translated into reports and other outputs. NLP, the technology allowing computers to understand and create human language (Eisenstein 2019), already allows a significant shift in how we can interact with large volumes of data. Rather than using the traditional methods of complex database queries, formulas, and statistical analysis, NLP can now facilitate users with minimal to no computer science or statistical knowledge to ask meaningful questions using their own words. This in turn can democratize access to the available data collected in zoos on a daily basis and making that data useful in day‐to‐day operations for animal care, curatorial, veterinary, and other staff who need access to quick and uncomplicated answers to guide their decision making.

AI is also changing how scientific research is carried out. AI‐enhanced tools to support literature reviews are already available and will enable reduced time and effort to complete these (for a review see Fabiano et al. [2024]). Some of these tools are already capable of searching large volumes of literature and offer relevant papers, as well as summarizing their content, or answering plain language questions based on the available scientific literature.

However, it is important to understand the limitations around these technologies, as well as to acknowledge that biases exist in these (for reviews pertaining to agriculture and healthcare settings, respectively, see Nazer et al. [2023] and Mayuravaani et al. [2024]). AI systems are limited by the data they were trained on and are susceptible for biases stemming from the training data and process, including the omission or incorrect identification of data. As an example, machine vision applications may find it difficult to operate in low‐light, high‐contrast, or very complex environments (see also the Case Study in this paper), all of which are common in zoos and aquariums (Zhang et al. 2024). There is also evidence, that AI‐supported professionals, especially less experienced ones, are more likely to make incorrect decisions when provided with an incorrect, but plausible, AI‐generated suggestion (Dratsch et al. 2023). Therefore, human expertise and oversight are going to continue to be key factors.

## 5 Conclusions

Development of AI technology and applications, as well as new, more powerful algorithms to support these, is fast and accelerating (Tang et al. 2020; Jiang et al. 2022). It is likely that AI will continue to become part of many, if not most, everyday technologies. It is, however, the zoo and aquarium community's task to support the development of specific software and hardware technologies adapted to the tasks required through day‐to‐day animal care, management, and broader welfare. A prerequisite for this is a good general understanding of AI by animal care professionals, veterinarians, animal behavior and welfare scientists, and others participating in the day‐to‐day care of animals. Strong organizational support, including support from internal IT departments and/or external providers, is also necessary. Conflict due to perceived or real increase of IT costs, IT security risks, and resource requirements to provide ongoing support for new technologies can hinder innovation. Purpose built or customized AI applications can provide advances in zoo animal welfare, but only with collaboration between the different experts necessary to develop these technologies further.

Undoubtedly, AI will become increasingly important and powerful, and sharing of experiences within the zoo community could speed up progress and reduce costs associated with development of different applications and technologies. The variety of species housed in zoos and the potential to customize AI for specific species make the sharing of experiences especially important. So does the potential lack of affordability of these systems for smaller, less wealthy organizations.

We propose that regional zoo organizations could act as catalyzers in this, providing platforms to share experiences and actual working solutions accumulated by their membership. Like working groups or specialist advisory groups exist for many areas within the zoo community, including for veterinary, conservation education, research, or field conservation, it would be wise to consider if technology or innovation focused groups could be introduced to provide support and knowledge sharing opportunities in this field. An example of this is the Association of Zoos and Aquariums Technology Scientific Advisory Group, but similar groups in other regions are currently missing, to the authors' knowledge.

## 6 Case Study

The authors deployed an AI system built on Microsoft's Azure Machine Learning Studio to analyze close circuit television (CCTV) footage of a meerkat enclosure. The enclosure was equipped with five high‐resolution cameras monitoring the environment constantly, generating 120 h of video footage a day. Analyzing this amount of data in the traditional way, with staff or volunteers reviewing the footage, was not a viable option due to resource limitations.

The system employed both off the shelf and bespoke software components achieving a purpose‐built ML pipeline. Video footage downloaded from the CCTV system was utilized as input with frame rate reduced and still images analyzed through different available computer vision modules. Meerkat detections were recorded, including their location within the frame and estimated reliability of the detection. These data were processed further within the pipeline to identify animals' proximity to each other (recorded as “interaction”), or to pre‐defined objects within the enclosure. Data was then visualized and presented in the format of a dashboard, utilizing the commercially available Microsoft PowerBI software's capabilities. The dashboard included heat map visualization of data overlayed on the field of view of the CCTV cameras.

The system could detect meerkats within the enclosure, though detection reliability depended on the used ML algorithm. A pre‐programmed animal classifier failed to identify meerkats when the animals were not in the species typical upright sentry position, showing a bias in the training data set utilized by this algorithm. A more generic computer vision model, MegaDetector, however, achieved high reliability in recognizing animals within the picture, whether moving or stationary, both in a sentry and quadrupedal position.

The development of the system took about 3 months using an agile software development methodology. Collaboration between the zoo's animal behavior and welfare staff, IT professionals providing general support to the zoo, as well as the software platform provider and software developers was key. The generated data allowed heatmap visualization of enclosure use, including the use of predefined enclosure furnishing, as well as 24/7 assessment of behavior, neither of which were previously feasible through keeper observations.

## Ethics Statement

The study described in the Case study section received ethical approval from the School of Veterinary Medicine and Science, University of Nottingham (4123 240509).

## Data Availability

Data sharing is not applicable to this article as no datasets were generated or analyzed during the current study.
